# From wires to waves, a novel sensor system for in vivo pressure monitoring

**DOI:** 10.1038/s41598-024-58019-5

**Published:** 2024-03-30

**Authors:** Daniel Nilsen Wright, Mark Züchner, Eis Annavini, Manuel J. Escalona, Lena Hammerlund Teige, Lars Geir Whist Tvedt, Andreas Lervik, Henning A. Haga, Thomas Guiho, Ingelin Clausen, Thomas Glott, Jean-Luc Boulland

**Affiliations:** 1 Department of Microsystems and Nanotechnology, SINTEF AS, Oslo, Norway; 2 Department of Neurosurgery, Oslo University Hospital, Oslo, Norway; 3 Division of Physiology, Department of Molecular Medicine, Institute of Basic Medical Sciences, University of Oslo, Sognsvannsveien 9, 0317 Oslo, Norway; 4 Department for Immunology, Clinic for Laboratory Medicine, Oslo University Hospital-Rikshospitalet, Sognsvannsveien 20, 0372 Oslo, Norway; 5 Department of Companion Animal Clinical Sciences, Norwegian University of Life Sciences, Ås, Norway; 6 CAMIN team, INRIA, Montpellier, France; 7 InVivo Bionics AS, Oslo, Norway; 8 Sunnaas Rehabilitation Hospital, Nesoddtangen, Norway

**Keywords:** Piezoresistive pressure sensor, MICS, Implant, Pig model, Wireless, Bluetooth, MEMS, Preclinical research, Implants

## Abstract

Pressure monitoring in various organs of the body is essential for appropriate diagnostic and therapeutic purposes. In almost all situations, monitoring is performed in a hospital setting. Technological advances not only promise to improve clinical pressure monitoring systems, but also engage toward the development of fully implantable systems in ambulatory patients. Such systems would not only provide longitudinal time monitoring to healthcare personnel, but also to the patient who could adjust their way-of-life in response to the measurements. In the past years, we have developed a new type of piezoresistive pressure sensor system. Different bench tests have demonstrated that it delivers precise and reliable pressure measurements in real-time. The potential of this system was confirmed by a continuous recording in a patient that lasted for almost a day. In the present study, we further characterized the functionality of this sensor system by conducting in vivo implantation experiments in nine female farm pigs. To get a step closer to a fully implantable system, we also adapted two different wireless communication solutions to the sensor system. The communication protocols are based on MICS (Medical Implant Communication System) and BLE (Bluetooth Low Energy) communication. As a proof-of-concept, implantation experiments in nine female pigs demonstrated the functionality of both systems, with a notable technical superiority of the BLE.

## Introduction

Pressure monitoring is a process during which measurements are continuously carried out for a defined duration, depending on the condition. This could range from a few minutes, to several years. Monitoring pressure in different organs of the body serves both diagnostic and therapeutic purposes. This application spans various medical disciplines, such as cardiology, neurology, ophthalmology, urology, and gastroenterology^1^. Pressure sensors have been employed in clinical settings for many years. The most common underlying principle involves transmitting the pressure from the target organ through a liquid or air medium to an external transducer. With basic engineering designs, it is possible to develop relatively reliable and cost-effective devices for external pressure recordings^2^. However, existing clinical equipment presents different limitations. Artifacts from patient movements are a recurrent problem with water-filled catheters, which can be improved by using air-charged catheters^3^. However, comparative measurements revealed that air-charged catheters provided significantly different readings when compared to water-filled catheters. An alternative approach involves using a microtip probe, where the pressure transducer is located at the tip of the probe^4^. This approach may reduce movement artifacts and solve the problem of clogged catheters. When compared with water pressure transducers in a mechanical bladder model, there was a high correlation in pressure readouts between the two systems^5^. However, clinical studies pointed out inconsistencies between these different systems^6,7^. Therefore, the latest international guidelines for optimal urodynamic practices, advocate the use of fluid-filled catheters with external transducer systems during cystometry^8,9^.

Optical fiber pressure sensor systems and microelectromechanical systems (MEMS) have emerged as promising technologies for a wide range of medical applications^10–15^. Among these devices, piezoresistive pressure sensors are a type of MEMS that convert mechanical pressure exerted on a membrane into changes in the electrical resistivity. One achievement in using MEMS technology for medical applications is the marketing of the CardioMEMS HF system for wireless monitoring of pulmonary arteries in heart failure patients^16^. In previous studies, we reported on the construction and characterization of a MEMS pressure sensor element (i.e., the pressure sensitive component) that could be utilized to develop new medical devices^17^. We also conducted bench tests using a bladder model on a vibrating table, which showed that this sensor could detect signals that current clinical systems are unable to detect^18^. Furthermore, a clinical case study using this sensor system suggested that it is safe to use and provides reliable measurements in the urinary bladder^19^.

Among others, patients with neurogenic urinary bladder—often caused by spinal cord injuries—would benefit from further development of wireless implantable sensor systems for prolonged measurements. This technology could replace the lost bladder-filling sensation by monitoring bladder pressure, offering a valuable source of information to the patient and the healthcare personnel. Hence, over the past decade, various prototypes of implantable pressure sensor systems have been developed, employing real-time telemetric links or data logging capabilities for later retrieval^20–31^. Different communication protocols, such as WIFI, ZigBee, Near Field Communication, Medical Implant Communication System (MICS), and Bluetooth Low Energy (BLE), can be implemented in such systems^32,33^.

In this study, we describe the iterative development of an implantable pressure sensor system based on a piezoresistive sensor element and probe from earlier studies^17^. Initially, we used a tethered data logger prototype designed as part of a diagnostic tool, which allowed for simultaneous data logging and real-time streaming. Later, we upgraded to an implantable wireless MICS communication system. To further improve the system performance, we designed an implantable BLE unit. To assess a new prototype of a medical device, it is customary to conduct benchtop tests or employ dummy anatomical models to demonstrate that the system operates as intended^18,25^. Ex vivo benchtop trials with a living isolated organ can also be performed. However, to obtain more reliable and comprehensive data, further implantation in an animal model is necessary. The selection of the animal model depends on the specific experimental requirements, such as the size of the implant and the type of the sensor system^34^. Using nine female pigs, which served as a widely recognized animal model for translational research^35^, we tested each of these systems in vivo. This enabled us to identify issues in the system's functionality and make changes to improve its performance. Taken together, this development resulted in sensor systems that provided reliable pressure measurements in organs with different pressure dynamics.

## Results

### Pressure recordings from a tethered sensor system

**Figure 1:**
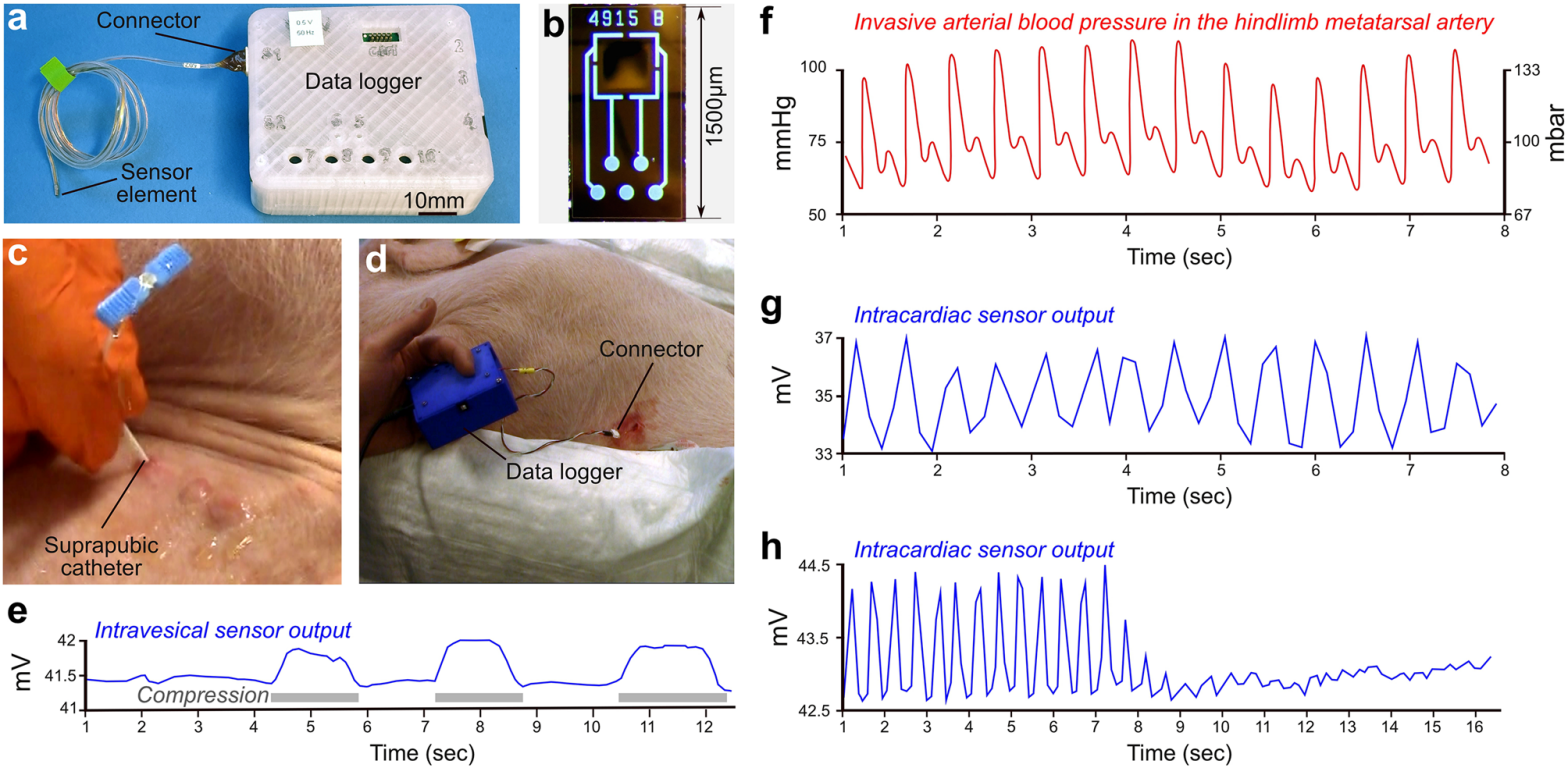
Test of the tethered sensor system in vivo*.* (**a**) Prototype of the tethered sensor system. It comprises a data logger connected to a sensor probe at the tip of which is mounted the sensor element (**b**) Sensor element. (**c**) Suprapubic catheter placement for implantation of the pressure sensor probe in the urinary bladder. (**d**) Transcardiac implantation of the sensor probe. (**e**) Response of the sensor system when pressure is manually applied to the lower abdominal area at the level of the urinary bladder. (**f**) Intraoperative monitoring of arterial blood pressure from the hindlimb metatarsal artery by a conventional clinical system. (**g**) Intracardiac pressure recording by the tethered sensor system. (**e**) Sensor output demonstrating the loss of intracardiac pressure upon sacrificing of the pig. Of note, (**g**) and (**h**) were obtained from experiments in different pigs, using distinct sensor probes.

This sensor system was composed of a data logger (Fig. 1a) connected to a soft sensor probe, at the tip of which the sensor element was mounted (Fig. 1b). During the in vivo tests, the sensor probe was implanted in the urinary bladder of pigs (n = 6) by a suprapubic approach. The correct placement of the pressure probe was confirmed with ultrasound and the presence of urine droplets from the cannula (Fig. 1c). To verify that pressure increase could be recorded by the sensor system, we performed repeated mechanical compressions on the lower abdomen of the pig, where the urinary bladder is situated. With each compression, the sensor system exhibited increased output values (Fig. 1e). For testing the system in a dynamic environment beyond the urinary bladder, we implanted the sensor in the hearts of two pigs, using a transthoracic approach targeting the left ventricle (Fig. 1d). Throughout the entire test period, which lasted at least 25 min, the sensor probe tolerated the dynamic environment and functioned as expected. As anticipated, the sensor output was synchronized with arterial blood pressure (Fig. 1f and g). Toward the end of the experiment, during the euthanasia of the pig (n = 2), the output of the pressure sensor system abruptly dropped to zero, coinciding with the occurrence of cardiac arrest (Fig. 1h).

### Assessment of the MICS system for in vivo recordings

**Figure 2:**
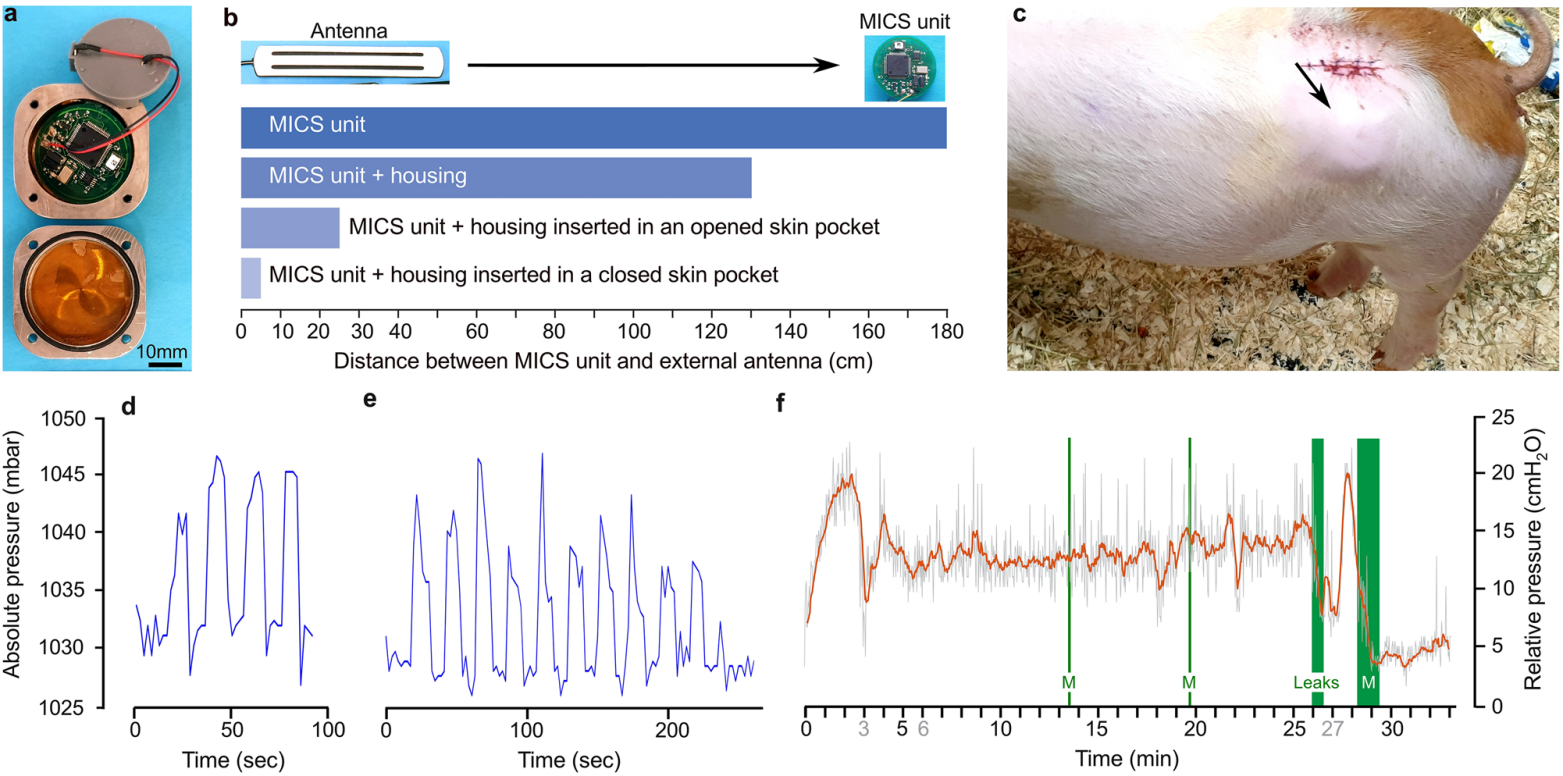
In vivo pressure recordings from the wireless MICS-system implanted in a pig. (**a**) MICS unit and the titanium housing. (**b**) Bar chart illustrating the maximum connection distance between the MICS unit and the base station under different conditions. (**c**) The titanium housing, indicated by the arrow, is subcutaneously implanted in the pig. (**d**) Pressure increase observed in response to manual squeeze of the urinary bladder during surgery. (**e**) Pressure increase observed in response to manual pressure applied to the lower part of the pig’s abdomen. (**f**) Pressure recording of a full bladder during the awakening process of the pig, after surgical anesthesia. M, micturition.

To progress toward a fully implantable system, we assembled the sensor probe with a wireless transceiver that was provided—as a courtesy—by Prof. Sawan (Sup. Fig. 1a–c). It was built on a 3-cm diameter PCB. As the transmission was based on the MICS protocol, we called it the MICS unit. The reliability of the complete system was confirmed through bench tests in a custom-made pressure chamber (Sup. Fig. 2a and b). The output voltage of the sensor system demonstrated consistency and reproducibility across multiple cycles, spanning from 800 to 1200 mbar (Sup. Fig. 2c). At the same time, this served as a calibration system for the sensor element, following a second-order polynomial fit (Sup. Fig. 2d). We also determined that the maximum connection distance between the MICS unit and the external antenna, in an open line in the air, was 1.8 m (Fig. 2b). The MICS unit was engineered to acquire and store data with low power consumption. We estimate an average current draw of 1.8 mA during data collection. However, upon user request, connection to the based station and data transfer consumed on average 5 mA with spikes at 17 mA. The memory buffer allows a maximum of 3710 data points for each of the two sensor channels. Assuming the fastest sampling rate of 0.5 Hz, equivalent to two hours of recording before transmitting data, the MICS unit should be able to operate for approximately 16 days on a battery capacity of 750 mAh.

To assess the performance of the wireless system in a living environment, acute experiments were conducted in pigs (n = 4). In these experiments, the sensor probe was implanted while the MICS unit remained externally positioned, allowing for potential troubleshooting (Sup. Fig. 3a). We successfully established a connection with the MICS unit to initiate and conclude the data logging process. To evaluate the response of the sensor system during urinary bladder contractions, we electrically stimulated the spinal sacral micturition center^36^. As a result, we observed an increase in urinary bladder pressure accompanied by a micturition event (“M” in Sup. Fig. 3b). After refilling the bladder through a suprapubic catheter and resuming the stimulation, we could elicit a second and more substantial increase in bladder pressure, followed by another micturition event.

As the next step was to achieve a fully implanted system, including the MICS unit, we designed a titanium housing and connectivity components that were water-resistant (Fig. 2a). Bench testing of this system revealed that the titanium housing reduced the maximum connection distance to 1.3 m (Fig. 2b). We then performed a full implantation of the complete system in a pig (n = 2). While the skin pocket accommodating the titanium housing was still open, the maximum connection distance was substantially decreased to 25 cm. Once the pocket was closed with sutures, the communication distance was further reduced to 5 cm (Fig. 2b and c). However, during this surgery, we obtained confirmation of the proper functioning of the sensor system. This is evidenced by the consistency of the pressure variation recorded by the system in response to gentle squeezing of the urinary bladder (Fig. 2d), and to the application of external pressure to the lower abdomen (Fig. 2e). In addition, during the pig’s awakening phase, several instances of micturition were observed (Fig. 2f). The logs revealed an initial rise in bladder pressure, which could be interpreted as a bladder contraction. It was followed by a second potential contraction, and two micturition events occurred at 13 and 20 min. In addition, we observed subsequent “leak” events, characterized by small intermittent voids. Other episodes, suggestive of bladder pressure variations not followed by micturition or leaks, could also be observed at 18 and 22 min. The nature of these episodes is difficult to determine; they could represent bladder contractions or could be artefacts. At 27 min, another possible bladder contraction was followed by an abundant micturition and a subsequent decrease in bladder pressure.

### Performance of the BLE system

Despite its ability to record physiological bladder events, there are several areas in the initial MICS prototype that could potentially be improved. These include enhancing the communication distance, possibly by improving the transmission strength or the antenna design. It might also benefit from a revision of the housing, using a material that has less shielding properties than titanium. Other improvements we aimed at were to increase the sampling rate, currently limited to every two seconds, and to implement real-time data streaming instead of saving data on the unit for later downloading. Although these latter two points were intended by design, we found that they were quite limiting during the in vivo experiments. Finally, we aimed to improve the power management despite these apparently more energy-demanding criteria. To address these aspects, we developed a novel communication system based on BLE, using a similar general architecture as for the MICS unit (Sup. Fig. 1a and d). We conducted bench tests like those performed for the MICS unit and obtained consistent outputs. The BLE unit achieved real-time transmission at a sampling rate of 8 Hz. This could be increased up to 128 Hz by adjusting the ADC bit depth. As expected, the power profiling showed that the highest power drain occurred during data transmission (Sup. Fig. 4). With an average current draw of 2.8 mA, and a 750 mAh battery, our calculations suggest that the system can operate continuously for around 11 days before the battery is depleted. We measured the maximum connection distance in an open corridor and found that it was at least 40 m.

**Figure 3:**
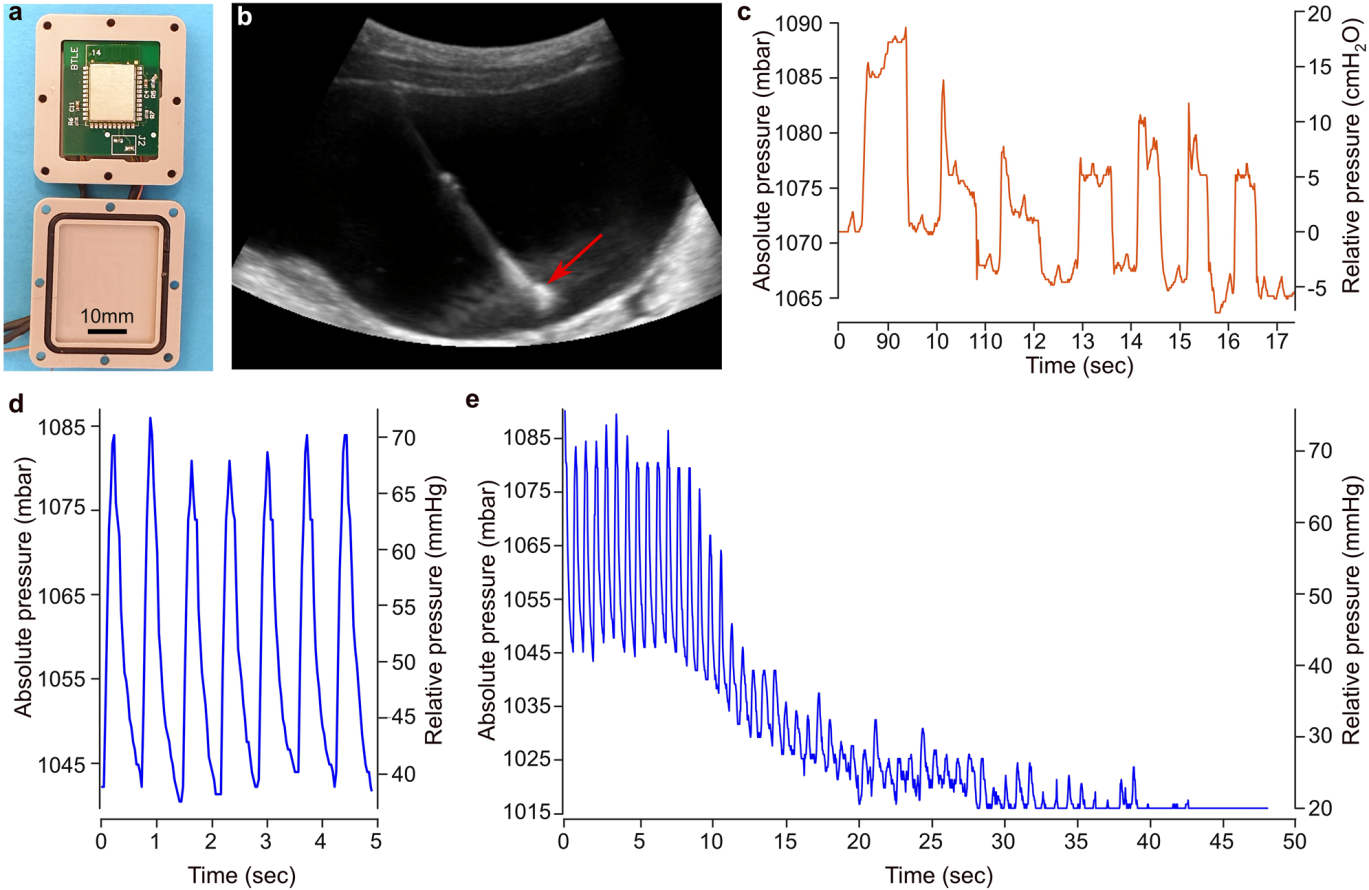
In vivo pressure recordings from the wireless BLE system implanted in a pig. (**a**) BLE unit placed in the PEEK housing. (**b**) Representative sonogram confirming the presence of the sensor probe in the pig’s urinary bladder. (**c**) Increase in bladder pressure observed in response to manual pressure applied to the pig’s abdomen. (**d**, **e**) Recording of arterial blood pressure from the femoral artery during the surgery (**d**) and at the time of the pig’s termination (**e**).

To further improve the radio frequency wave propagation from the implanted unit to the external receiver, we created the housing for the BLE unit using PEEK (polyether ether ketone) instead of titanium (Fig. 3a). Once the sensor probe was implanted in the urinary bladder (Fig. 3b), the BLE unit was placed under the skin and we determined that maximum distance for wireless connection was eight meters, with a signal strength of –83 dBm, which falls within the operational range for the receiver. The application of extracorporeal pressure to the lower part of the abdomen systematically increased the read-out of the bladder pressure (Fig. 3c). Since the BLE unit has a higher sampling rate compared to the MICS unit, it provided the opportunity to test the sensor system in a dynamic and high-pressure environment. Consequently, we implanted the sensor in the femoral artery for a duration of 2 h and 45 min. The sensor signal, sampled at different time points remained consistent throughout this period. Furthermore, we tested the signal quality at different sampling rates. While the signals were relatively similar for 16 Hz and 32 Hz, there was a clear degradation at 128 Hz (Sup. Fig. 5a) consistent with the change in the resolution of the ADC; while at the lowest sampling rate (8 Hz) the ADC has a 16 bit resolution, its resolution is reduced to 12 bits at 128 Hz. An autocorrelation of the signal at 32 Hz showed a strong rhythmic pattern, characterized by a coefficient of 1 at lag 0, along with recurring peaks and valleys at regular lags, which also exhibited high coefficient values (Sup. Fig. 5b). The frequency of the signal was calculated at 1.45 Hz, which matched the frequency recorded by the intraoperative blood pressure monitoring device. In this challenging environment, the system remained functional throughout the entire period and continued to operate after cleaning the sensor element and later bench tests. Owing to technical difficulties, we were unable to perform a full system calibration prior to the implantation in the pig. Nonetheless, a post-experimental calibration allowed us to calculate the amplitude of the signal. We obtained blood pressure readings at 73/40 mmHg. This contrasted with the measurements obtained from the blood pressure monitoring device, at 84/45 mmHg. At the end of the experiment, during the pig’s euthanasia, the BLE system recorded a blood pressure drop that eventually reached a flat line (Fig. 3e), consistent with the readings from the blood pressure monitoring device.

## Discussion

Advances in MEMS technology provide new opportunities for monitoring pressure in various organs of the body. The recording of a piezoresistive pressure sensor probe in a bladder of a human for 17 h shows that this new type of medical device is on the doorstep of clinical practice^19^. Our results during in vivo implantation experiments in different organs of the pig further support the translational potential of this type of technology. Because piezoresistive sensors have the potential to be implanted in different tissues to record pressure variations in multiple conditions^15,37^, we implanted our prototype in organs that have different pressure dynamics. The compliance of the urinary bladder, on account of its stretchable tissue, allows for the storage of a large volume of urine at low pressure^38^. Therefore, after implanting the pressure sensor probe in the bladder, the sensor readout was found to be linear and stable. However, the sensor system demonstrated responsiveness to manual application of external pressure on the lower abdomen, indicating its functionality. Unlike the urinary bladder, the heart produces vigorous contractions with rapid pressure changes that propagate to the arteries. Consequently, we implanted the pressure sensor probe in the heart and arteries to investigate the opposite end of the pressure spectrum. Because the tethered data logger was not intended for this use, it operated with a sampling rate that was insufficient to capture a detailed curve. As for the MICS unit, its sampling rate did not allow to perform this test. In contrast, a clear blood pressure curve could be obtained with the BLE system at both 16 Hz and 32 Hz. This allowed us to precisely calculate the heart rate and show that it was identical to what was detected by the intraoperative blood pressure monitoring system, confirming the reliability of this prototype.

An important objective in this development was to create a fully implantable prototype. Such a medical implant could significantly advance health monitoring in various medical conditions like cardiovascular diseases^30^ and lower urinary tract disorders^39,40^. To bring our prototype closer to this goal, we initially tried a wireless data communication system that operates within the MICS bandwidth. While the integrated circuit achieved was relatively compact, the addition of the battery, its holder, and the titanium housing resulted in a complete unit that was thicker than desired. The implantation in pigs confirmed the functionality of the system and micturition could be correlated with bladder pressure increases although the pig was still partially under the influence of the anesthesia, which affects bladder physiology. However, there were several aspects of this prototype that could be improved. The initial low power consumption strategy of the MICS, which consisted of establishing only two telemetric connections at the start and the end of the measurement, was abandoned. This is because the animal experiments showed that a more flexible system that includes real-time monitoring was beneficial. Therefore, we designed a new system based on BLE communication that maintains a permanent communication link with the external receiver. One substantial improvement of this was the increased sampling rate, which allowed us to target organs characterized by dynamic pressure changes, like the femoral artery in a pig. While we have demonstrated the reliability of this system through precise heart rate measurement, the calculation of the blood pressure differed from the values provided by the blood pressure monitoring system. The systolic and diastolic blood pressures were 13% and 11% lower than expected. This apparent discrepancy may be because the sensor probes used for testing the BLE had previously been used in a human clinical trial. Another explanation could be that for technical reasons, we were unable to calibrate the sensors prior to the implantation. As a potential sensor drift may have occurred during experiment, and the sensor probe removal and cleaning, the use of a post-implantation calibration curve to fit the blood pressure measurement may be inadequate.

In the future, we will continue the development of the BLE unit. Safety circuits, such as a current limiter, will have to be introduced^41^. Despite these additional circuits, the size of this unit could be reduced by optimizing the design of the circuit board, and using electronic components that have smaller packaging (e.g. the Bluetooth communicator). Another important aspect is to reduce power consumption, which would, in turn, allow the reduction of battery size for a more compact system. A considerable source of power drain comes from data transmission, and thus power can be saved by reducing the frequency of communication to a minimum. The Bluetooth standard allows for a maximum interval of 32 s before disconnecting the central from the peripheral. Longer intervals between data transmissions can be achieved by exploiting the ability of a peripheral to quickly reconnect to a central it had previously connected to, a process known as direct advertising. This strategy would require accumulating data for as long as possible, then reconnecting and transmitting, thereby pushing the transmission interval to about 4 min. While this method has the potential to significantly cut down on power usage, it comes with the trade-off of losing real-time streaming capabilities. A potential solution could be to introduce a feature that allows for switching between these two data acquisition modes based on user requirements. The sensor element represents another source of continuous, albeit lower, power consumption. Therefore, power could also be saved by optimizing the power supply to the sensor element. Reducing the transmission strength to the antenna could also be a possible way to save power. The BLE communication distance was tested to be at least 40 m in open space, but it decreased to 8 m after implantation. Reducing this distance to 3 or 4 m when implanted might suffice. This could be adjusted in the software by the end-user. Another possible power management strategy could be to use smaller size rechargeable batteries or a battery-less system, as previously done^27,31,42^. In our system, the battery is placed under the skin. This allows the use of large enough batteries to reduce the need for frequent recharges without compromising on signal sampling rate or continuity of monitoring. Other improvements to the system could include the encapsulation of the BLE unit, which is crucial to ensure that it is impermeably sealed to isolate it from body fluids while maintaining data transmission. Several biocompatible materials can fulfill this requirement^43,44^. Importantly, the choice of material, thickness, communication protocol, and antenna design can significantly affect the degree of signal attenuation. Encapsulation in silicone and other biocompatible polymers is a relatively simple and cost-effective process. However, over time, these materials tend to allow water permeation. Titanium housing, commonly used in various electronic medical implants such as electrical simulators, was initially chosen as the MICS protocol is known to pass through a thin layer of this material^45^. However, our experience in working with titanium is that it is time-consuming, expensive, and requires specific equipment. Hence, for the BLE unit, we used PEEK, which is cheap, easy to work with, biocompatible, autoclavable, and allows for fast development. Future prototypes may also take advantage of 3D printing technology using biocompatible material.

Altogether, the performance and flexibility of the BLE unit exceeds the two former prototypes and will constitute the basis for further development. Such a system could contribute to pressure measurements in a variety of clinical conditions, both as a semi-implanted device for short clinical examinations and as an implant for long-term monitoring.

## Materials and methods

### Design of the sensor element and sensor packaging

A piezoresistive sensor element was designed and manufactured by SINTEF^17^. In brief, it comprises a 2-µm thick single-crystalline silicon device layer with a complete Wheatstone bridge configuration. This device layer is anodically bonded to a glass wafer containing cavities located beneath the pressure sensing membranes^46^. The sensor element has been seamlessly integrated into a probe, and the materials exposed to the environment comply with biocompatibility standards, such as USP Class VI and/or ISO-10993. Further information about the sensor probe can be found in a separate publication^19^.

### Engineering of the SDL, MICS and BLE units

The engineering of the tethered sensor data logger (SDL) was previously reported^19^. Briefly, it consists of the assembly of an analog digital converter, a microcontroller, and a micro-SD card. The SDL is powered by batteries or through a USB connection to a PC. The measurement data is stored on the micro-SD card, while a software interface allows for real-time streaming and local storage of the data. Unfortunately, during acute implantation experiments in pigs, the SDL data files were corrupted several times. The data presented in Fig. 1 could however be salvaged thanks to video recordings that were later digitized.

For the engineering of the implantable wireless units, MICS and BLE, an overview of their system architecture is presented in Supplementary Fig. 1a. The signal from the sensor is amplified, digitized, and stored on a low power sensor interface. Both systems were built with small commercial off-the-shelf components.

The MICS unit was obtained—as a courtesy—from the laboratory of Prof. Sawan, Department of Electrical Engineering, Polytechnique Montréal. This system is based on a MICS component powered by a battery and capable of communicating with an external base station (Sup. Fig. 1b). The assembly and communication modules were from Microsemi Corporation (Arizona, USA) conforming to the MICS standard. Four prototypes were built on a round PCB resulting in a unit of 30 mm in diameter (Sup. Fig. 1c). The MICS unit is connected to a 30-mm coin battery holder to accommodate a Panasonic CR2477 1000 mAh lithium battery. A LabVIEW (National Instruments, Texas, USA) program with a graphical user interface allows the retrieval of the data stored in the memory and the configuration of the MICS unit.

Four prototypes of the BLE unit were manually assembled on a 30 × 25 mm PCB. The Bluetooth stack ran on an MK13A module from MOKOBlue, which is based on a Nordic Semiconductor nRF5340 SOC, while analog-to-digital conversion was performed using two Texas Instruments ADS1100 ADCs. The whole system was powered by a 750 mAh lithium thionyl chloride EF651625 battery from EVE Energy Co, regulated through a Linear Technology LTC3544 quad buck regulator. Data collection was handled by a custom Python script using an nRF52840DK Development Kit as a receiver unit.

### Sensor system characterization and calibration

The complete wireless sensor systems (with MICS/BLE communication means) underwent characterization and calibration. To perform the calibration, the sensor probe and MICS/BLE unit were placed inside a dedicated aluminum pressure chamber (Sup. Fig. 2a and 2b). This chamber was designed to withstand pressure and was connected to a Fluke 6270A pressure controller and calibrator via a 6 mm hose. Inside the chamber, a fixture was specifically designed and 3D-printed to support up to four sensor probes and the MICS/BLE unit. The tip of the sensor probe was immersed in water during the characterization. The chamber was then closed and partially submerged in a thermally controlled water bath set at 37 °C. Pressure was applied in increments of 25 mbar, ranging from 800 to 1200 mbar and back to 800 mbar. A representative calibration curve is presented in supplementary Fig. 2c. Using these data points, a calibration equation was derived through polynomial fitting of the pressure versus the averaged sensor output for each pressure level (Sup. Fig. 2d). This equation was subsequently used to calculate the pressure values from the sensor outputs recorded during the animal trials. Of note, for the BLE in vivo experiment, the two sensors implanted—in the bladder and the femoral artery—could not be calibrated before the implantation due to unforeseen technical issues and time constraints. Therefore, the plots presented in Fig. 3 were created using a calibration curve obtained after the implantation. The sensors were removed, cleaned with saline, dried, and underwent calibration a few days later. To compensate for value drift that may have occurred during this process, the blood pressure curve was normalized to the pressure recorded by the intraoperative monitoring system after termination of the pig. For the bladder pressure measurements, normalization was not done. Due to the production costs associated with the sensor elements and packaging, we opted to reuse sensors from a previous clinical trial.

### Housings for in vivo implantation

To be fully implantable, the MICS/BLE units had to be isolated from body fluids in a manner that did not compromise data transmission. To build the housing of the MICS unit, we used titanium. Water resistance was ensured by a rubber joint at the interface of the two pieces (Fig. 2a). The top and bottom walls thinned down to 500 µm thin. The housing was assembled with four corner M2.5 T8 screws. A hole was drilled for the cable to pass through the wall of the housing. Silicon rubber (Smooth-On, Inc, Macungie, PA) and epoxy (EPO-TEK 353ND-T) were applied on the inside and the outside of the housing to maintain water resistance. For the housing of the BLE unit, the overall design was similar, although there were differences in dimensions and materials used (Fig. 3a). Instead of titanium, we utilized PEEK (polyether ether ketone), a biocompatible plastic material that supports BLE transmission. To test the water resistance, the housings were immersed in a container filled with colored water.

### Animals

Animal experiments were carried out at the Section for Experimental Biomedicine at the Department of Production Animal Clinical Sciences (ProdMed), at the Norwegian University of Life Sciences (NMBU), SEARCH at NMBU, and the Centre for Clinical, Experimental Surgery, and Translational Research of the Biomedical Research Foundation of the Academy of Athens. These facilities are approved by EU standards and regulations to carry out animal experiments on large animals. In Norway, the procedure was approved by the Norwegian Animal Research Authority (*Forsøksdyrutvalget*) under the identification numbers 7089 and 19,435. In Greece, the research was conducted in compliance with the legal requirements for animal experimentation, in harmonization to the European Directive 2010/63, and it was approved by the Veterinary Service of the Prefecture of Athens under registration number 5522/24-10-2018. In compliance with the FELASA guidelines, all efforts were made to minimize the number of animals used and their suffering. This report has been written using the ARRIVE guidelines for animal research. All surgical procedures, post-operative procedures, and euthanasia were supervised by veterinarians. In total, we used nine female pigs weighing between 25 and 50 kg. Eight of them had a genetic background consisting of 50% Norwegian land pig, 25% Norwegian Yorkshire, and 25% Duroc, and one had a genetic background of 50% Landrace and 50% Large White female pigs. This inconsistency was a consequence of the COVID-19 pandemic; however, it is not expected to have any impact on the results.

### Anesthesia and analgesia

Food was withheld from pigs 12 h prior to the induction of anesthesia. Anesthesia for acute experiments was performed as previously described^47^. For short-term survival experiments, the pigs were premedicated with dexmedetomidine 80 µg/kg (0.5 mg/ml Dexdomitor, Orion) and midazolam 0.6 mg/kg (5 mg/ml Dormicum, Roche) intramuscularly. An auricular vein was catheterized prior to induction of anesthesia by propofol 1.2 mg/kg intravenously. The pigs were endotracheally intubated and connected to an anesthetic machine. Anesthesia was maintained by isoflurane in a mixture of oxygen and air and positive pressure ventilation was applied. An arterial catheter was placed in the hindlimb metatarsal artery for intraoperative monitoring of blood pressure. After zeroing the pressure transducer, it was positioned at the level of the heart. Prior to recovery, ketoprofen 3 mg/kg and buprenorphine 20 µg/kg were administered intravenously. Lidocaine 20 mg/ml 10 ml was infiltrated into the pocket containing the titanium housing. In all pigs, vital signs were monitored using an anesthetic monitor.

### Surgical implantation

For acute experiments, an ultrasound echo sonogram was performed to locate the targeted organs. For urinary bladder implantation, the sensor probe was inserted with suprapubic technique, using a T-peel introducer needle (I-Flow corporation, Lake Forest, CA 92630, USA). A similar approach was used for intracardiac implantation. Correct placement was also checked by withdrawal of urine or blood. The sensor probe was externally connected to either the tethered data logger or the MICS/BLE unit.

Two pilot survival experiments were also performed during which the urinary bladder was solely targeted. The first approach was suprapubic, as described earlier. Two skin incisions were performed on the hypogastric (2 cm, midline) and left iliac abdominal (5 cm) regions. A channeling tube was used to create a subcutaneous tunnel between the two incisions. After suprapubic insertion of the sensor probe, the wire and connectors were led subcutaneously to the abdominal iliac incision through the channeling tube. The hypogastric incision was closed. A subcutaneous pocket was created in the lumbar region (rostral to the left middle gluteal muscle), to accommodate the housing. The channeling tube was used to subcutaneously tunnel the sensor probe between the lumbar and the left iliac abdominal incision and connect it to the titanium housing. A silicon rubber mixture (Smooth-On, Inc, Macungie, PA) was applied to the connectors to ensure water resistance. The silicon rubber block and the titanium housing were sterilized by immersion in a Rely + On Perasafe (Puls AS, Norway) solution for 15 min and rinsed with saline before implantation. The wounds were sutured, and local anesthetic was administered subcutaneously. The second approach was inspired from^48^. To secure the sensor probe in the urinary bladder, a 3-way Foley balloon catheter was attached with suture stitches. A subcutaneous pocket to accommodate the MICS unit was created, as described earlier. A low midline abdominal incision was performed to expose the urinary bladder. The urinary bladder was identified and a small incision of about 5 mm was performed on the ventral bladder wall to insert the catheter and the sensor probe attached to it. The balloon was then inflated with 2 ml sterile saline and the bladder incision was closed using a 3-0 Propylene suture. A channeling probe was used to tunnel the extension cable from the subcutaneous pocket accommodating the housing, through the abdominal wall to connect the MICS unit with the sensor probe. The connection was embedded and sterilized, as explained earlier. The abdominal wall was sutured in 3 layers, the pocket wound was sutured, and local anesthetic was administered subcutaneously.

### Spinal cord stimulation

For some acute experiments, the sensor system was used to record bladder pressures while attempting to induce stimulation-driven bladder contractions. Spinal cord stimulation at a sacral level was implemented after performing a sacral laminectomy to expose the L5-S3 spinal cord. Stimulations targeting the sacral micturition center were delivered using a monopolar electrode placed on the dorsal surface of the spinal cord. Trains of biphasic balanced cathodic-first pulses of electrical currents with variable intensity were delivered using a wearable neural stimulator—called STIMEP—designed in compliance with EU directives for active medical devices (90/385, 93/342 and EN 62304 before the application of the new EU regulation MDR2017/745) by the CAMIN team (INRIA/University of Montpellier, France) in association with the Axonic company (Sophia Antipolis, Vallauris, France)^36^.

### Post-operative care

Cephalosporin (25 mg/kg, Zinacef, GSK) was administered at the beginning and by the end of the procedure. Ketoprofen was administered i.m. for 2 days after the surgery. The pigs were monitored, and the wound areas were palpated to check for pain at least twice daily. Rescue analgesia in the form of Buprenorfin was available, but not required.

### Termination

For the acute experiments, the pigs were euthanized while in anesthesia with an i.v. injection of potassium chloride. For one survival experiment, one pig was stunned using a captive bolt gun, while for the other, the pig was sedated as described earlier and euthanized with a 10 ml bolus dose (i.v.) of sodium pentobarbital (400 mg/ml Exagon, Richter Pharma).

### Supplementary Information

Supplementary Figures.

## Data Availability

The datasets used and/or analysed during the current study is available from the corresponding author on reasonable request.
