# Supplementary material for: From wires to waves, a novel sensor system for in vivo pressure monitoring
Source: Sci Rep. 2024 Mar 30;14:7570. doi: 10.1038/s41598-024-58019-5 (PMC10981663; doi:10.1038/s41598-024-58019-5)
Supplement: Supplementary file 1 — Supplementary Figures. [file 41598_2024_58019_MOESM1_ESM.pdf]

# From wires to waves, a novel sensor system for in vivo pressure monitoring

Daniel N. Wright[1,#], Mark Züchner[2,3,#], Eis Annavini[3], Manuel J. Escalona[3,4], Lena H. Teige[3,4], Lars Geir Whist Tvedt[1,7], Andreas Lervik[5], Henning A. Haga[5], Thomas Guiho[6], Ingelin Clausen[1,7], Thomas Glott[8], Jean-Luc Boulland[3,4]*

[1]Department of Microsystems and Nanotechnology, SINTEF Digital, Oslo, Norway
[2]Department of Neurosurgery, Oslo University Hospital, Norway
[3]Section of Physiology, Department of Molecular Medicine, Institute of Basic Medical Sciences, University of Oslo, Norway
[4]Department for Immunology, Oslo University Hospital, Norway
[5]Department of Companion Animal Clinical Sciences, Norwegian University of Life Sciences, Ås, Norway
[6]CAMIN team, INRIA, Montpellier, France
[7]InVivo Bionics AS, Norway
[8] Sunnaas Rehabilitation Hospital, Nesoddtangen, Norway
[#]Contributed equally

*Correspondence: Jean-Luc Boulland (j.l.boulland@medisin.uio.no)

## Supplementary information

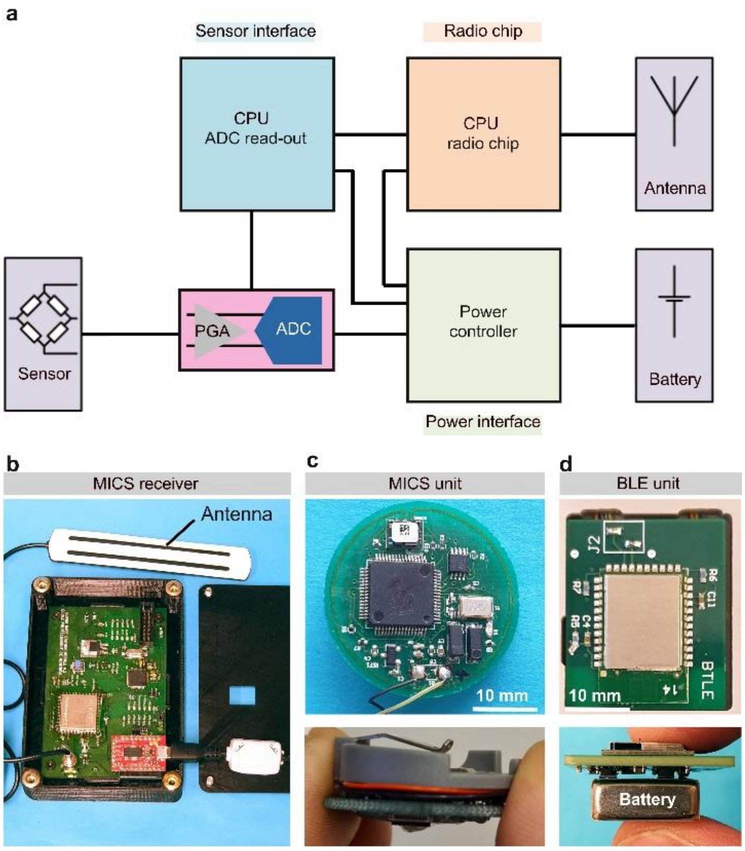


**Supplementary Figure 1:** Engineering of the MICS and the BLE units (a). Overview of the system architecture. This is a revised diagram based on the original document provided by Prof. Sawan, as a courtesy. (b) MICS base station. (c) MICS unit. (d) BLE unit.

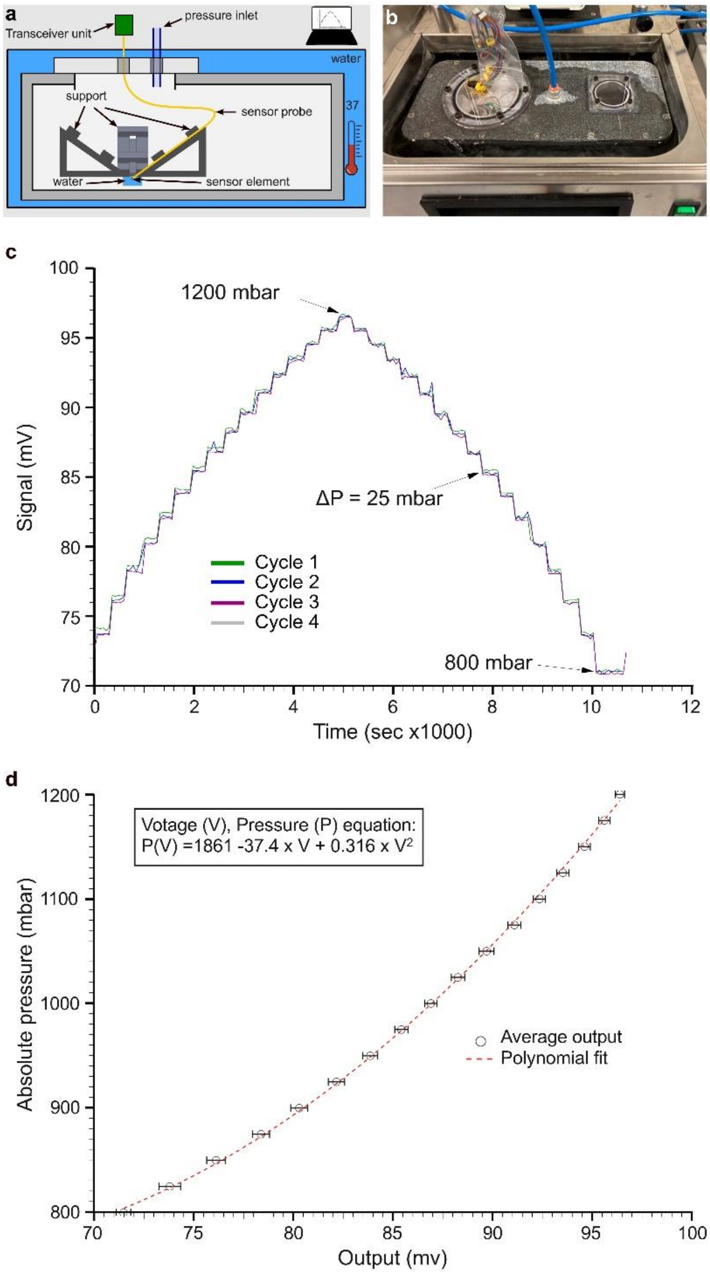


**Supplementary Figure 2:** Sensor system characterization and calibration. (a) Schematic diagram illustrating a pressure chamber designed to accommodate a holder for up to four sensor probes. The sensor element is submerged in water at the bottom of the chamber. (b) Photograph of the actual chamber described in (a). A pressure line (blue) and a pressure controller allow for adjusting the pressure inside the chamber. The chamber can be immersed in a thermally controlled water bath for temperature control. (c) Characterization curve of a sensor system using the chamber depicted in (a and b), using pressure steps of 25 mbar. (d) Calibration curve: Pressure versus averaged sensor output curve. The calibration equation is a second-order polynomial.

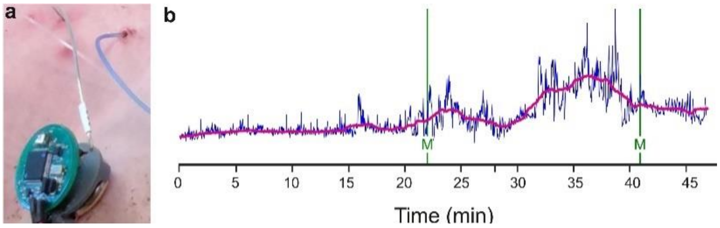


**Supplementary Figure 3:** In vivo semi-implantation of the MICS wireless sensor system. (a) The sensor probe is implanted while the MICS unit is left outside the body. (b) Stimulation of the sacral spinal cord leads to an increased sensor output.

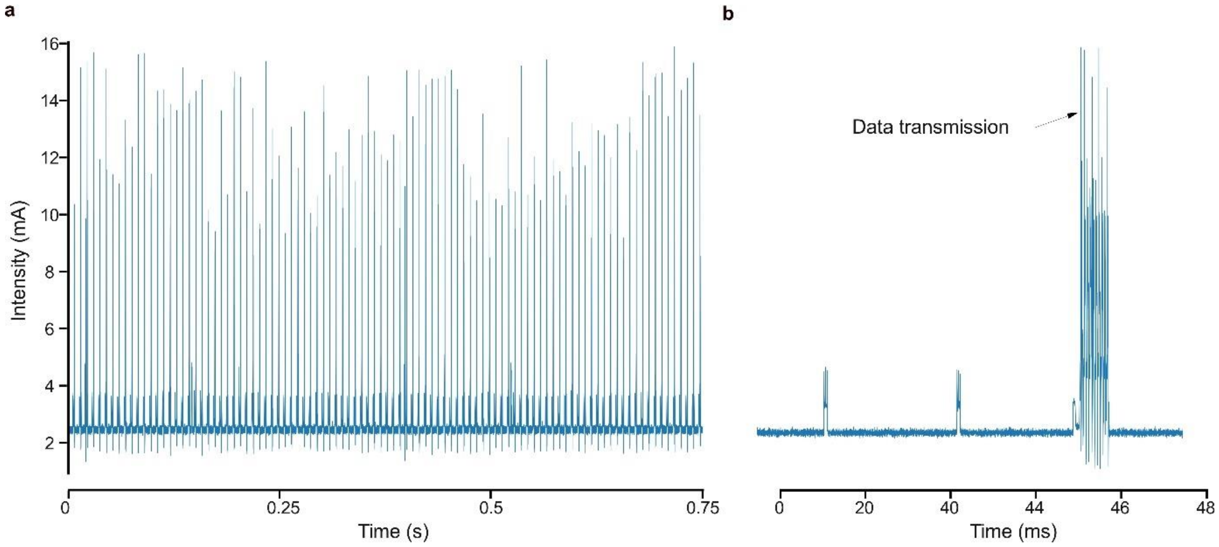


**Supplementary Figure 4:** Power consumption test of the BLE unit (a) Power drawn by the BLE unit during data acquisition and real-time transmission (b) Magnified plot from (a).

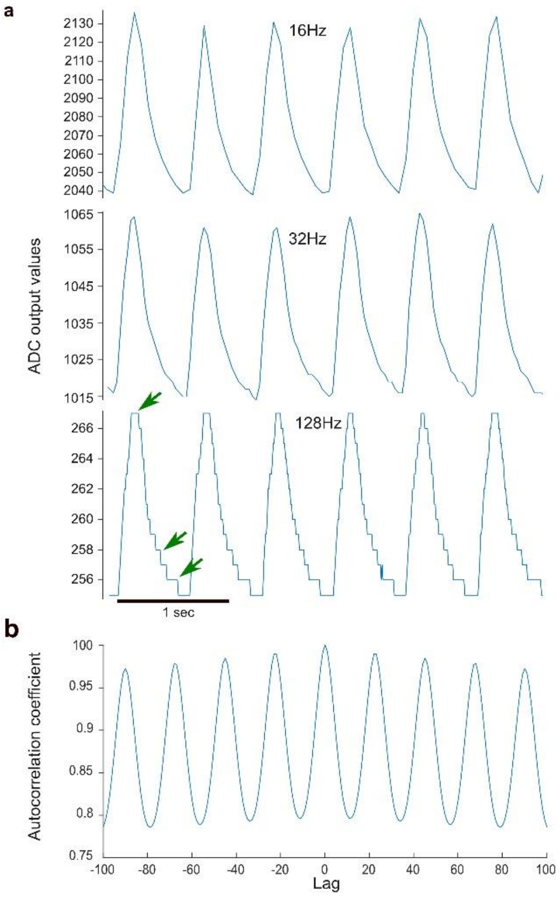


**Supplementary Figure 5:** Characterization of the arterial blood pressure data received from the BLE unit. (a) Test of three different data sampling rates: 16 Hz, 32 Hz, and 128 Hz. (b) Autocorrelogram obtained from a 32 Hz data sample.
